# 表达IL7和CCL19的第四代CD19 CAR-T联合PD-1单抗治疗复发难治大B细胞淋巴瘤的疗效和安全性

**DOI:** 10.3760/cma.j.issn.0253-2727.2023.10.005

**Published:** 2023-10

**Authors:** 腾 俞, 辉 刘, 文 雷, 攀攀 陈, 爱琪 赵, 相贵 袁, 基民 高, 文斌 钱

**Affiliations:** 1 浙江大学医学院附属第二医院血液内科，杭州 310009 Department of Hematology, the Second Affiliated Hospital, College of Medicine, Zhejiang University, Hangzhou 310009, China; 2 温州医科大学检验医学院，温州 325035 Wenzhou Medical University Laboratory Medicine, Wenzhou 325035, China

**Keywords:** 嵌合抗原受体T细胞, 大B细胞淋巴瘤, 免疫检查点抑制剂, 免疫相关不良反应, Chimeric antigen receptor T cells, Large B-cell lymphoma, Immune checkpoint inhibitor, Immune related adverse reaction

## Abstract

**目的:**

系统性研究表达白细胞介素7（IL7）和趋化因子C-C基序配体19（CCL19）的第四代靶向CD19嵌合抗原受体T细胞（CD19 CAR-T）联合PD-1单抗治疗复发难治大B细胞淋巴瘤的疗效及安全性。

**方法:**

应用自体7×19 CAR-T联合替雷利珠单抗治疗11例复发难治大B细胞淋巴瘤患者，评估其疗效及不良反应。

**结果:**

11例入组患者均完成自体7×19 CAR-T的制备和回输，9例患者完成预定的6次替雷利珠单抗治疗，1例患者完成4次，1例患者完成1次。5例（45.5％）达完全缓解，3例（27.3％）达部分缓解，客观缓解率72.7％。2例评估为疾病进展，1例在回输后2个月因疾病不能控制而死亡。中位随访时间31（2～34）个月，中位总生存时间未达到，中位无进展生存时间为28（1～34）个月，2例部分缓解患者分别在随访第9个月和第12个月进一步获得完全缓解，故最佳完全缓解率为63.6％。细胞因子释放综合征和免疫效应细胞相关神经毒性综合征均可控，未见免疫相关不良反应发生。

**结论:**

自体7×19 CAR-T联合替雷利珠单抗治疗复发难治大B细胞淋巴瘤取得较好疗效，且不良反应可控。

## Abstract (en)

**Objective:**

This study systematically explore the efficacy and safety of fourth-generation chimeric antigen receptor T-cells（CAR-T）, which express interleukin 7（IL7）and chemokine C-C motif ligand 19（CCL19）and target CD19, in relapsed or refractory large B-cell lymphoma.

**Methods:**

Our center applied autologous 7×19 CAR-T combined with tirelizumab to treat 11 patients with relapsed or refractory large B-cell lymphoma. The efficacy and adverse effects were explored.

**Results:**

All 11 enrolled patients completed autologous 7×19 CAR-T preparation and infusion. Nine patients completed the scheduled six sessions of tirolizumab treatment, one completed four sessions, and one completed one session. Furthermore, five cases（45.5％）achieved complete remission, and three cases（27.3％）achieved partial remission with an objective remission rate of 72.7％. Two cases were evaluated for disease progression, and one died two months after reinfusion because of uncontrollable disease. The median follow-up time was 31（2−34）months, with a median overall survival not achieved and a median progression-free survival of 28（1−34）months. Two patients with partial remission achieved complete remission at the 9th and 12th months of follow-up. Therefore, the best complete remission rate was 63.6％. Cytokine-release syndrome and immune effector cell-associated neurotoxicity syndrome were controllable, and no immune-related adverse reactions occurred.

**Conclusion:**

Autologous 7×19 CAR-T combined with tirelizumab for treating relapsed or refractory large B-cell lymphoma achieved good efficacy with controllable adverse reactions.

第四代嵌合抗原受体T（CAR-T）细胞表达免疫刺激细胞因子如白细胞介素7（IL7）、IL15或IL12等，以及趋化因子如趋化因子C-C基序配体19（C-C motif chemokine ligand 19，CCL19）等，能够提升CAR-T持久性、对肿瘤细胞靶向性和杀伤能力，因此又称装甲化CAR-T[1]–[4]。我们团队构建以CD19为靶点，共表达IL7和CCL19的第四代CAR-T，将其命名为7×19 CAR-T，注册Ⅰb期多中心临床试验，结果表明其在复发难治大B细胞淋巴瘤治疗中具有较好安全性和疗效，但仍有部分患者复发或难治，部分原因可能是淋巴瘤微环境（lymphoma microenvironment, LME）免疫检查点抑制，而程序性死亡受体1（PD-1）及其配体上调也在CAR-T耗竭中发挥了重要作用[5]–[6]。以PD-1单抗阻断免疫检查点提高CAR-T疗效，已经在临床前研究和个案中展现出应用前景，但两者联合方式缺乏系统性研究，是否会引发严重细胞因子释放综合征（CRS）、免疫效应细胞相关神经毒性综合征（ICANS）或引发额外的免疫相关不良反应（immune-related adverse event, irAE）也需进一步探索[7]–[8]。故在本研究中，本中心应用自体7×19 CAR-T联合替雷利珠单抗（一种人源化IgG4抗PD-1单克隆抗体）治疗复发难治大B细胞淋巴瘤患者，现就其有效性和安全性报道如下。

## 病例与方法

1. 病例资料：纳入2020年7月至2021年5月于浙江大学医学院附属第二医院进行7×19 CAR-T联合替雷利珠单抗治疗的复发难治大B细胞淋巴瘤患者11例。所有患者在入组前均接受过2线以上治疗，疾病复发或难治，并存在可评估病灶，美国东部肿瘤协作组（ECOG）体能状态评分0～3分，合并慢性乙型病毒性肝炎患者需接受抗乙肝病毒治疗且乙肝病毒DNA检测阴性，其余入排标准同ZUMA-1研究[9]。本研究经浙江大学医学院附属第二医院伦理委员会批准，批件号：（2021）伦审研第（0531）号，所有患者均充分知情并签署知情同意书。

2. CAR-T制备及回输：本研究所用的7×19 CAR-T为第四代CAR-T，以4-1BB为共刺激分子，并含有IL7和CCL19表达序列，均由本中心专职人员负责制备，制备过程在符合GMP标准的实验室完成。制备的基本流程如下：患者签署知情同意书后，采集外周血T淋巴细胞，在体外通过慢病毒载体将CAR片段转导入T细胞，中位转导率为30％（7％～58％）。在CAR-T输注前给予氟达拉滨联合环磷酰胺方案清淋预处理化疗，具体剂量为氟达拉滨30 mg·m^−2^·d^−1^，环磷酰胺500 mg·m^−2^·d^−1^，均为−5 d～−3 d连续3 d给药；替雷利珠单抗在CAR-T回输后31 d开始，每21 d静脉滴注给药1次，每次200 mg共计6次，具体流程见图1。

**图1:**
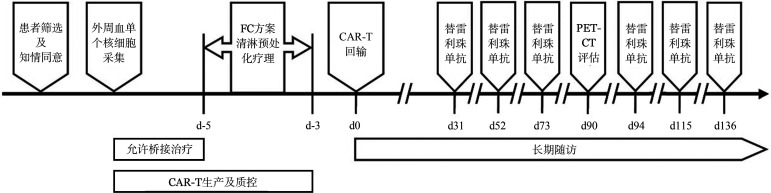
自体7×19 CAR-T联合替雷利珠单抗治疗复发难治大B细胞淋巴瘤流程图 注 FC：氟达拉滨联合环磷酰胺

3. 疗效评估：CAR-T回输后90 d行全身PET-CT评估疗效，根据Lugano淋巴瘤疗效评价标准执行，分为完全缓解（CR）、部分缓解（PR）、疾病稳定（SD）和疾病进展（PD），客观缓解率（ORR）定义为CR率+PR率[10]。同时评估最佳CR（best complete response，bCR），bCR率定义为在长期随访中获得的最佳疗效为CR的比例。

4. 不良反应评估：不良反应重点关注CRS和ICANS，按照美国移植与细胞治疗学会（American society for transplantation and cellular therapy，ASTCT）共识进行分级[11]。而长期随访中irAE根据美国卫生和公共服务部发布的常见不良反应术语评定标准（CTCAE）5.0进行分级和管理。

5. 随访：通过门诊或住院复查，以B超及CT作为定期疗效评估手段，随访时间截至2023年7月1日。总生存（OS）时间定义为CAR-T回输时间至任何原因导致死亡；去进展生存（PFS）时间定义为CAR-T回输时间至疾病进展或死亡。

6. 统计学处理：应用IBM SPSS 20版本软件进行统计学分析。计数资料以例数（百分比）表示，计量资料以M（范围）表示，通过Kaplan-Meier生存分析计算OS及PFS；通过Reverse Kaplan-Meier法计算中位随访时间。

## 结果

1. 一般临床特征：共纳入11例复发难治患者，其中男9例，女2例，中位年龄50（40～70）岁。弥漫大B细胞淋巴瘤（DLBCL）9例（其中含EB病毒阳性DLBCL 1例），转化滤泡性淋巴瘤（transformed follicular lymphoma，TFL）1例，伴有MYC和BCL2重排的高级别B细胞淋巴瘤（high-grade B-cell lymphoma，HGBL）1例。患者既往接受治疗中位线数为3（2～6）线，接受治疗中位次数为11（5～33）次。2例患者接受过自体造血干细胞移植，1例患者接受累及野放射治疗，7例患者接受BTK抑制剂治疗，2例患者接受BCL2抑制剂维奈克拉治疗。其余各项临床特征详见表1。

**表1:** 11例复发难治弥漫大B细胞淋巴瘤患者的临床特征

临床特征	例数	构成比（%）
性别		
男	9	81.8
女	2	18.2
年龄		
≤60岁	8	72.7
>60岁	3	27.3
诊断		
DLBCL	9	81.8
TFL	1	9.1
HGBL	1	9.1
B症状		
存在	3	27.3
不存在	8	72.7
ECOG体能状态评分		
0～1	6	54.5
2～3	5	45.5
Ann Arbor分期		
Ⅰ～Ⅱ期	6	54.5
Ⅲ～Ⅳ期	5	45.5
结外受累		
≤1处	8	72.7
>1处	3	27.3
乳酸脱氢酶		
正常	1	9.1
升高	10	90.9
疾病状态		
难治	9	81.8
复发	2	18.2
IPI评分		
0～1	3	27.3
2	3	27.3
3	2	18.2
4～5	3	27.3
既往化疗		
2线	1	9.1
3线	5	45.5
4线	2	18.2
5线及以上	3	27.3

注 DLBCL：弥漫大B细胞淋巴瘤；TFL：转化滤泡性淋巴瘤；HGBL：高级别B细胞淋巴瘤；IPI：国际预后指数；ECOG：美国东部肿瘤协作组

2. 7×19 CAR-T治疗：从细胞采集到7×19 CAR-T回输的中位时间为14（12～15）d，期间2例患者接受糖皮质激素作为桥接治疗。中位回输剂量为2（1～3）×10^6^/kg。9例患者完成预定的6次替雷利珠单抗治疗，1例患者完成4次，1例患者完成1次，均因疾病进展提前终止PD-1单抗治疗。CAR-T回输后90 d全身PET-CT评估提示，5例（45.5％）达CR，3例（27.3％）达PR，ORR为72.7％。2例经PET-CT评估为PD，1例在回输后2个月因疾病不能控制而死亡。中位随访时间31（2～34）个月，中位OS时间未达到，中位PFS时间为28（1～34）个月，2例PR患者分别在随访第9个月和第12个月进一步获得CR，故bCR率为63.6％，详见图2。

**图2:**
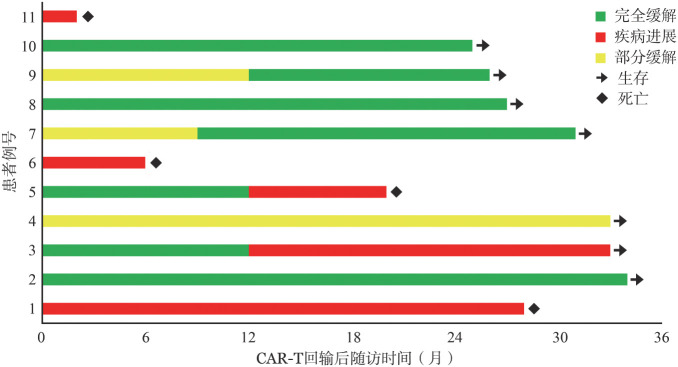
11例接受表达IL7和CCL19的CD19 CAR-T联合替雷利珠单抗治疗的复发难治大B细胞淋巴瘤患者生存情况

3. 不良反应：11例入组患者输注CAR-T后10例患者发生不同程度CRS反应，出现CRS的中位时间为输注后第2（1～8）d，其中1级CRS 3例，仅表现为发热，以补液和对症处理后好转，2级CRS 5例，3级CRS 2例，表现为发热、低血压或低氧血症，通过托珠单抗及糖皮质激素治疗好转，CRS中位持续时间为10.5（4～14）d。2例患者发生1级ICANS，表现为头痛，1例患者经评估达到3级ICANS，表现为头痛、定向力下降、语言能力和书写能力下降，经糖皮质激素应用和对症处理后好转。后续随访中未见irAE事件发生。

## 讨论

第四代7×19 CAR-T治疗复发难治大B细胞淋巴瘤，本中心已经注册Ⅰb期多中心临床试验，入组39例复发难治大B细胞淋巴瘤，3个月PET-CT疗效评估提示ORR为79.5％，其中CR率56.4％，中位随访时间32个月，中位PFS时间为13个月，中位OS时间未到达。尽管7×19 CAR-T通过改进 CAR-T 扩增和促进肿瘤组织浸润的机制，较第二代 CAR-T 的疗效有所提高，但仍有部分患者复发或难治，可能与CAR-T耗竭或肿瘤微环境抑制因素等有关[5]。

肿瘤微环境免疫检查点信号紊乱，尤其PD-1及其配体，抑制CAR-T杀伤能力，诱导CAR-T耗竭，从而导致CAR-T治疗失败。有研究表明，PD-1过表达T细胞所制备的CAR-T抗肿瘤免疫反应差[12]。JULIET研究显示，PD-1与其配体相互作用评分较高的DLBCL患者接受CAR-T治疗无效，或在缓解后短时间内复发[13]。为克服PD-1信号通路紊乱，通过阻断PD-1以提升CAR-T疗效成为研究的热点。国内学者研究揭示，PD-1单抗治疗有助于共刺激域为4-1BB的CD8^+^ CAR-T细胞向中心记忆型细胞定向分化，促进CAR-T在体内维持和长期抗肿瘤作用[14]。另有学者构建表达PD-1阻断蛋白的CAR-T，通过体外实验和动物实验证明其能逆转肿瘤细胞对CAR-T的耐药[15]。

因此本研究应用替雷利珠单抗，阻断PD-1信号通路，以此联合自体7×19 CAR-T治疗复发难治大B细胞淋巴瘤患者，探索安全性和有效性。CAR-T回输后90 d全身PET-CT评估提示，ORR为72.7％，CR率为45.5％，2例PR患者分别在随访第9个月和第12个月进一步获得CR，故bCR率为63.6％，对照既往单用自体7×19 CAR-T的研究未见短期疗效显著提升。分析原因在于较既往单用研究，本研究入组患者治疗更为后线，且接受BTK抑制剂、BCL2抑制剂等靶向治疗的比例更高；有2例患者因疾病进展而没有完成既定的6次替雷利珠单抗治疗，这也是部分原因。进一步随访发现，中位随访时间31个月，中位OS时间未达到，中位PFS时间为28个月，较既往单用研究，在相似的中位随访时间里，观察到中位PFS的大幅度改善。且值得关注的是，2例3个月疗效评估为PR的患者，在完成全部6次替雷利珠单抗单抗，未使用其他治疗的情况下，分别在随访第9个月和第12个月进一步获得CR，使本研究bCR率提升至63.6％，提示阻断PD-1信号通路可能有助于提升CAR-T疗效。

本研究也表明CRS和ICANS可逆，远期随访也未见irAE发生，提示联合治疗安全性可控。但仍有部分患者在获得CR后出现疾病再次进展和复发，虽然后续通过更换新靶点CAR-T或维泊妥珠单抗等新的治疗，进一步延长了生存，但也提示单独阻断PD-1信号通路可能仍不足以逆转CAR-T难治或耐药，亟须新的联合策略，比如BCL2抑制剂或BTK抑制剂联合策略等[16]。

近期研究也表明，肿瘤微环境中CAR-T的抑制性因素并不局限于PD-1信号通路，包括T细胞免疫球蛋白黏蛋白分子3（T cell immunoglobulin and mucin-containing molecule 3, TIM-3）、淋巴细胞激活基因（lymphocyte activation gene 3, LAG3）和T细胞免疫球蛋白和ITIM结构域（T cell immunoreceptor with Ig and ITIM domain, TIGIT）等免疫检查点均对CAR-T发挥负调控效应[17]。因此在未来，对多种免疫检查点实施联合阻断策略也是一种极具潜力的CAR-T疗效提升方法。

总之，自体7×19 CAR-T联合替雷利珠单抗治疗复发难治大B细胞淋巴瘤具有较好的安全性和疗效。但由于研究样本有限，有待进一步扩大样本量，延长随访时间以提供更充足证据。
